# Th17/IL-17A Might Play a Protective Role in Chronic Lymphocytic Leukemia Immunity

**DOI:** 10.1371/journal.pone.0078091

**Published:** 2013-11-01

**Authors:** Iwona Hus, Agnieszka Bojarska-Junak, Sylwia Chocholska, Waldemar Tomczak, Justyna Woś, Anna Dmoszyńska, Jacek Roliński

**Affiliations:** 1 Department of Haematooncology and Bone Marrow Transplantation, Medical University of Lublin, Lublin, Poland; 2 Department of Clinical Immunology, Medical University of Lublin, Lublin, Poland; New York University, United States of America

## Abstract

Th17 cells, a recently discovered subset of T helper cells that secrete IL-17A, can affect the inflammation process autoimmune and cancer diseases development. The purpose of this study was to evaluate the role of Th17 cells and IL17A in biology of CLL. The study group included 294 untreated CLL patients in different clinical stages. Here, we show that higher Th17 and IL-17A values were associated with less advanced clinical stage of CLL. Th17 cells’ percentages in PB were lower in patients who died due to CLL during follow-up due to CLL (as compared to surviving patients) and in patients responding to first-line therapy with fludarabine-based regimens (as compared to non-responders). IL-17A inversely correlated with the time from CLL diagnosis to the start of therapy and was lower in patients who required treatment during follow-up. Th-17 and IL-17A values were lower in patients with adverse prognostic factors (17p and 11q deletion, CD38 and ZAP-70 expression). CLL patients with detectable IL-17A mRNA in T cells were in Rai Stage 0 and negative for both ZAP-70 and CD38 expression. Th17 percentages positively correlated with iNKT and adversely with Treg cells. The results of this study suggest that Th17 may play a beneficial role in CLL immunity.

## Introduction

Many issues of the development and progression of chronic lymphocytic leukemia (CLL), the most common leukemia diagnosed in adults in Western societies, still remain unclear. Complex immune disorders, present even in patients in early clinical stages, are one of the characteristic features of CLL and are considered to play an important role in disease pathogenesis. The natural history of CLL is extremely variable, with survival times from initial diagnosis that range from 2 to 20 years. Some patients die rapidly, within two to three years from diagnosis, other patients live for 10 to 15 years. Over 35 different prognostic markers have been tested and most found to be of limited, in predicting the course of nonadvanced stages of CLL. Some of these are classified as “traditional”, whereas others are classified as “novel”. The four novel prognostic markers that currently are in widespread use in clinical practice are the following: immunoglobulin heavy-chain variable region (IgVH) mutational status, interphase fluorescence in-situ hybridization (iFISH) abnormalities, CD38, and zeta-associated protein (ZAP)-70 [1].

These prognostic factors concentrate on the intrinsic abnormalities of the malignant B cell clone. It became clear that transformation and progression of tumors is not an independent process but it is controlled by their interactions with the tumor microenvironment [2]. The occurrence of several T cell abnormalities in patients with CLL is well studied and the changes in number and functions of T lymphocytes may support the “microenvironment” that sustains the malignant B cells clone, delays their apoptosis and may contribute to the pathogenesis and progression of the disease [3], [4], [5]. CD4+ T cells, especially the Th2 population, are involved in CLL progression [6]. In recent years, two new subsets of CD4 cells have been discovered, T regulatory and Th17 cells. T regulatory cells (Treg) are responsible for immune tolerance to tumor development and contribute to tumor growth in most cancer types [7], [8], [9], as well as in CLL [10], [11]. The role of Th17 cells in cancer has not been thoroughly explored, and appears to be divergent and controversial. Th17 cells were described as a distinct subpopulation of CD4+ T cells in 2005 and named after IL-17A, their hallmark cytokine. IL-17A exerts pleiotropic effects on nonimmune and immune cells and is an important mediator of the inflammation process [12]. It was documented that Th17 cells are important in the immune response against extracellular bacteria, various fungi, and viruses [13]. However, they are also involved in the development of a number of organ-specific autoimmune diseases and chronic inflammatory syndromes [14]. Their role in cancer development remains elusive. Data from experimental animal studies and from cancer patients suggest that Th17 cells may suppress or promote tumor growth [15], [16]. The role of IL-17A and Th17 cells in CLL immunopathogenesis remains undefined. Jain et al. reported the elevated numbers of Th17 cells in peripheral blood of patients with CLL and their association with favorable prognosis [17]. In this study, we further investigated the potential role of Th17 cells in chronic lymphocytic leukemia by analyzing the frequencies of Th17 cells and the level of IL-17A in peripheral blood (PB) and bone marrow (BM) of CLL patients in correlation with clinical and laboratory parameters characterizing both disease activity as well as patients’ immune status.

## Materials and Methods

### Ethic Statement

This study was approved by the Ethics Committee of the Medical University of Lublin (No. KE-0254/176/2009). Written informed consent was obtained from all patients.

### Patients and Samples

PB samples were obtained from 294 consecutive patients diagnosed with CLL in the Department of Hematooncology and Bone Marrow of the Medical University of Lublin. There were 162 women and 132 men, with the median age of 64 years (range 38–87). BM samples were obtained from 40 out of the 294 study patients. CLL diagnosis of was based on the criteria from the International Workshop on Chronic Lymphocytic Leukemia (IWCLL) [18]. Disease staging was determined according to the Rai classification system [19]. Patient characteristics at the time of CLL diagnosis are summarized in Table 1. All PB and BM samples were taken just after diagnosis and before the start of any anticancer therapy. Control PB samples were obtained from 45 healthy volunteers (HV) (25 women and 20 men, aged 32–70 years, median age of 59 years).

**Table 1:** Characteristics of the patients at CLL diagnosis.

Characteristics	No of patients (%)
Rai Stage	
0	91 (31.49)
I	61 (21.11)
II	86 (29.76
III	17 (5.88)
IV	34 (11.76)
ZAP-70 (cut-off 20%)	
Positive	112 (38.10)
Negative	182 (61.90)
CD38 (cut-off 20%)	
Positive	110 (37.41)
Negative	184 (62.59)
Cytogenetic abnormalities	
del(17p13.1)	9 (3.06)
del(11q22.3)	22 (7.48)
del(17p13.1) and del(11q22.3)	1 (0.34)
Without del(17p13.1) and del(11q22.3)	92 (31.29)
Not evaluated	170 (57.80)
WBC (G/L)	Median 28.9 (range: 1.70–528)
Hgb (g/dl)	Median 13.1 (range: 3.9–17.3)
Platelets (G/L)	Median 171 (range: 4–826)
LDH (U/L)	Median 366 (range: 176–1362)
B2M (mg/dL)	Median 2.69 (range: 1.8–22.0)

PB and BM samples were collected into heparinized tubes and immediately processed. Mononuclear cells were separated by density gradient centrifugation with Biocoll Separating Solution (Biochrom) for 25 minutes at 400×*g* at room temperature. Interphase cells were removed, washed twice, and resuspended in phosphate-buffered saline (PBS). PB and BM samples collected into EDTA tubes were used for plasma separation. Plasma samples were stored at –70°C until the time of analysis.

### Intracellular IL-17A Staining

**Figure 1:**
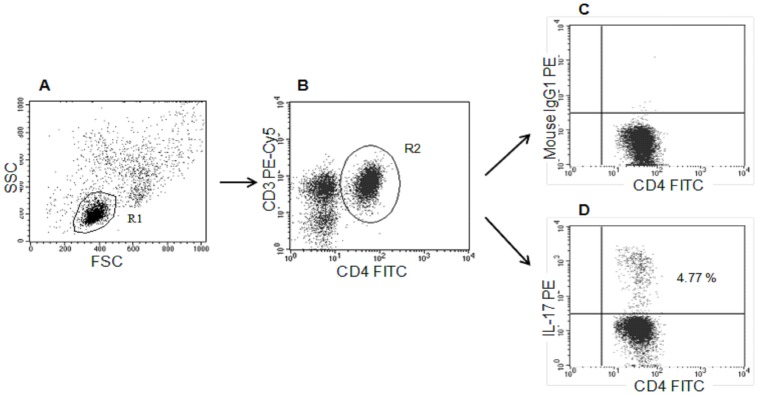
Flow cytometric analysis of Th17 cells. The dot plots show representative data from healthy control subjects, illustrating the analysis method for identification of CD4/CD3+/IL-17A+ cells (Th17). An acquisition gate was established basing on FSC and SSC that included mononuclear cells. A region, R1, was drawn around the lymphocytes (A). Next, the R1 gated events were analyzed for CD3 PE-Cy5 and CD4 FITC staining and positive cells (CD4+/CD3+) were gated (region R2) (B). The final dot plots CD4FITC versus mouse IgG1 PE (C) and CD4FITC versus IL-17A PE (D) were established by combined gating of events using R1 and R2. The number in the upper right quadrant on the dot plot D represents the percentage of CD4+/CD3+/IL-17A+ (Th17) cells.

Intracellular IL-17A analysis was performed on fresh PB samples from 150 CLL patients and 45 HV. Additionally, intracellular IL-17A expression was examined in BM samples from 40 CLL patients. Mononuclear cells (2×10^6^/ml) were cultured in RPMI 1640 supplemented with 2 mmol/L L-glutamine, 5% human albumin, 100 U/ml penicillin, and 100 µg/ml streptomycin. Cells were stimulated with 25 ng/ml of PMA and 1 µg/ml of ionomycin (Sigma, Germany) in the presence of BD GolgiStop (BD Pharmingen, USA) for 5 hours at 37°C in a 5% CO_2_ atmosphere. Cultured cells were washed twice in PBS, divided into tubes, and then stained with monoclonal antibodies (MoAb) against the cell-surface markers CD4 FITC and CD3 PE-Cy5 (BD Pharmingen). Following membrane staining, cells were fixed and permeabilized using Cytofix/Cytoperm Fixation/Permeabilization Kit (BD Pharmingen) according to the manufacturer’s instructions. Cells were then intracellularly stained with PE conjugated anti-IL-17A antibodies (BD Pharmingen) or a PE IgG1 isotype control. Finally, cells were washed and analyzed by flow cytometry, performed on a BD FACSCalibur System. Five data parameters were acquired and stored: linear forward and side scatter (FSC, SSC), log FL-1 (FITC), log FL-2 (PE), and log FL-3 (PE-Cy5). For each analysis, 20,000 events were acquired and analyzed using CellQuest Pro software. Isotype-matched antibodies were used to verify the staining specificity and as a guide for setting the markers to delineate positive and negative populations. Dot plots, illustrating the analysis method for the identification of CD4^+^/CD3^+^/IL-17A^+^ (Th17) cells are shown in Figure 1.

### Intracellular TNF, IL-10, and IL-4 Staining

Analysis of intracellular TNF, IL-10, and IL-4 expression by CD3+CD4+ cells was performed on fresh PB samples [20]. MoAbs used for analyses included anti-CD4 FITC, anti-CD3 PE-Cy5, anti-IL-10 PE (BD Pharmingen), anti-TNF PE, and anti-IL-4 (BD Biosciences). In the experiment, the mean percentages of CD3+CD4+ cells with intracellular cytokine expression were analyzed.

### Analysis of T Regulatory Cells

T regulatory cells were evaluated in the PB of 100 patients via analysis of the surface expression of CD4 and CD25 antigens, as well as intracellular expression of FoxP3 by flow cytometry. The percentage of CD4+CD25+FoxP3+ T regulatory cells (Treg) among CD4+ lymphocytes was determined using the Human Treg Flow Kit (FOXP3 Alexa Fluor 488/CD4 PECy5/CD25 PE) from BioLegend (San Diego, CA, USA) according to the manufacturer’s instructions.

### Assessment of iNKT Cells

Flow cytometry analysis of iNKT cells was performed in fresh PB samples of 100 patients using MoAbs anti-iNKT FITC (anti-Vα24 FITC) and anti-CD3 PE (BD Pharmingen). A standard, whole-blood assay with erythrocyte cell lysis was used for preparing the PB specimens. The samples were analyzed by flow cytometry directly after preparation.

### Flow Cytometric Analysis of CD38 and ZAP-70 Expression in CLL Cells

CLL cells were stained for CD38 antigen and ZAP-70 protein expression (as described previously [21]). The cut-off point for CD38 or ZAP-70 positivity in leukemic cells was ≥20%.

### Plasma IL-17A, TNF, and IL-10 Immunoassay

A commercial enzyme-linked immunosorbent assay (ELISA) kit (Quanticine Human IL-17 Immunoassay; R&D Systems) was used for a quantitative determination of human IL-17A in plasma samples. Likewise, commercial ELISA kits (Quanticine High Sensitivity Human TNF-Immunoassay and Quanticine human IL-10; R&D Systems) were used for a quantitative determination of human TNF and human IL-10 in plasma samples. Protocols followed were in accordance with the manufacturer’s recommendations. The ELISA Reader Victor^TM^3 (PerkinElmer, USA) was used.

### RNA Preparation and Quantitative ‘Real-time’ Reverse Transcription-polymerase Chain Reaction (qRT-PCR) for IL-17A

Expression of IL-17A mRNAs in CD4+ T cells was analyzed in samples obtained from 84 CLL patients. In 12 representative CLL patients, IL-17A mRNA expression in CD4+ cells was analyzed after PMA and ionomycin stimulation.

For RT-PCR, total RNA was isolated using the QIAamp RNA Blood Mini Kit (QIAGEN) from CD4+ cells. The CD4+ cells were purified by positive magnetic selection using CD4 MicroBeads (Miltenyi Biotec). RNA quantity was measured using a spectrophotometer (Bio-Rad). RNA was transcribed into cDNA using the QuantiTect Reverse Transcription kit (QIAGEN) according to the manufacturer’s protocol.

Real-time PCR was performed using TaqMan reagents specific for human IL-17A and internal control GAPDH (Applied Biosystems). The real-time PCR reactions were run for 40 cycles using universal cycling conditions (95°C for 10 minutes followed by 40 cycles at 95°C for 15 seconds and 60°C for 1 minute) on an Applied Biosystems 7300 Real-Time PCR System. Data was normalized to GAPDH (endogenous control). Data was analyzed using the threshold cycle (C_T_) and was presented as 2^−ΔCT^. ΔC_T_ is the difference between the C_T_ of the target gene (C_Tt_) and the reference gene (C_Tr_) (ΔC_T_ = C_Tt_ – C_Tr_).

### I-FISH Analysis

PB mononuclear cells were cultivated for 24 hours in RPMI 1640 medium without mitogen stimulation. After hypotonic treatment and methanol – acetic acid 3∶1 fixation, cell suspensions were dropped onto microscopic slides and used directly for I-FISH. The commercially available Vysis probes (Abbott Molecular Europe, Wiesbaden, Germany) LSI ATM SpectrumOrange/CEP 11 SpectrumGreen Probe and LSI TP53 SpectrumOrange/CEP 17 SpectrumGreen Probe were used. At least 200 nuclei were analyzed for each probe. The cut-off levels for positive values for normal controls were 2.5% (mean ± SD).

### Statistical Analysis

The Mann-Whitney U test was applied for statistical comparison of the results between CLL patients and HV, as well as between CLL patients in different stages of the disease. Comparisons among 3 or more groups were done with the Kruskal-Wallis test. The Wilcoxon paired test was used to compare the results in PB and BM. The Spearman rank correlation coefficient was used in correlation tests. The Kaplan-Meier method was used for drawing survival curves and results were compared using the log-rank test. The time from CLL diagnosis to the start of therapy was calculated from the date of diagnosis to the date of the first treatment. Statistical analyses were performed with STATISTICA 9.0 PL and Graphpad Prism 5 (Graphpad Software, Inc.). Differences were considered statistically significant with a p-value ≤0.05.

## Results

### Plasma IL-17A Level

**Figure 2:**
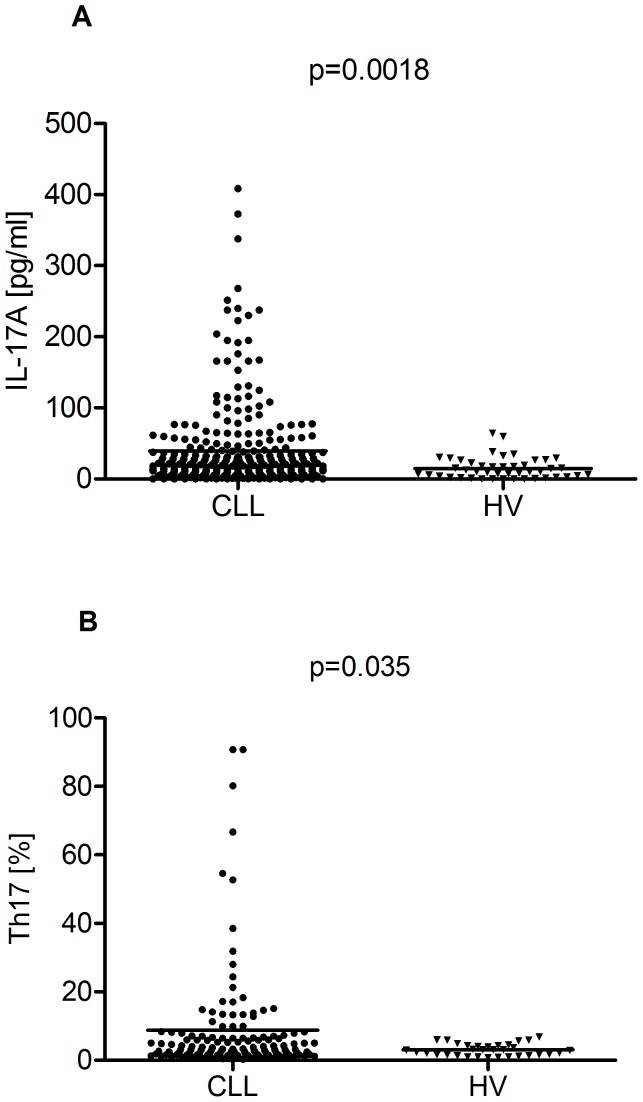
Plasma levels of IL-17A and percentage of Th17 cells in CLL patients and HVs. (A) The IL-17A concentration in PB from CLL patients an healthy volunteers (HVs) (39.35 pg/ml vs. 14.94 pg/ml, p = 0.0018). (B) The percentage of CD4^+^/CD3^+^/IL-17A^+^ (Th17) cells in CLL and HVs (8.76% vs. 3.02%, p = 0.035).

**Figure 3:**
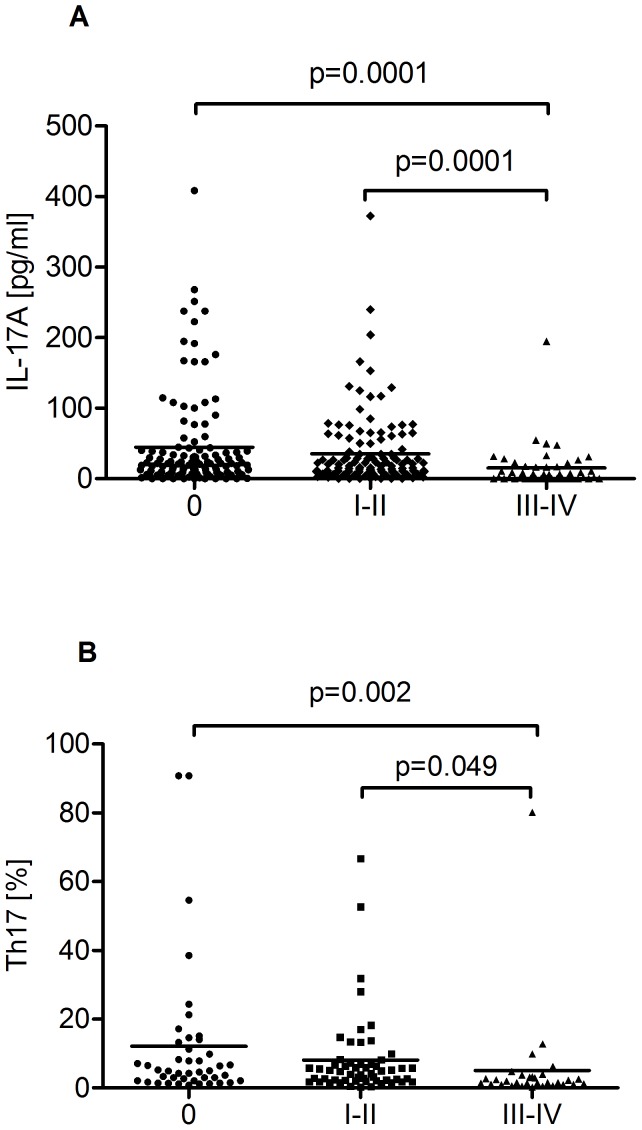
Plasma IL-17A levels and percentage of Th17 cells in CLL patients in different disease stages. (A) Plasma levels of IL-17A in CLL patients with Rai stages: 0 (median: 44.63 pg/ml), I–II (median: 35.33 pg/ml) and III–IV (median: 15.23 pg/ml). (B) Median percentage of Th17 cells in CLL patients with Rai stage 0 (median, 12.11%) in comparison to those in Rai stages I–II (median, 8.11%) and III–IV (median, 5.15%).

**Figure 4:**
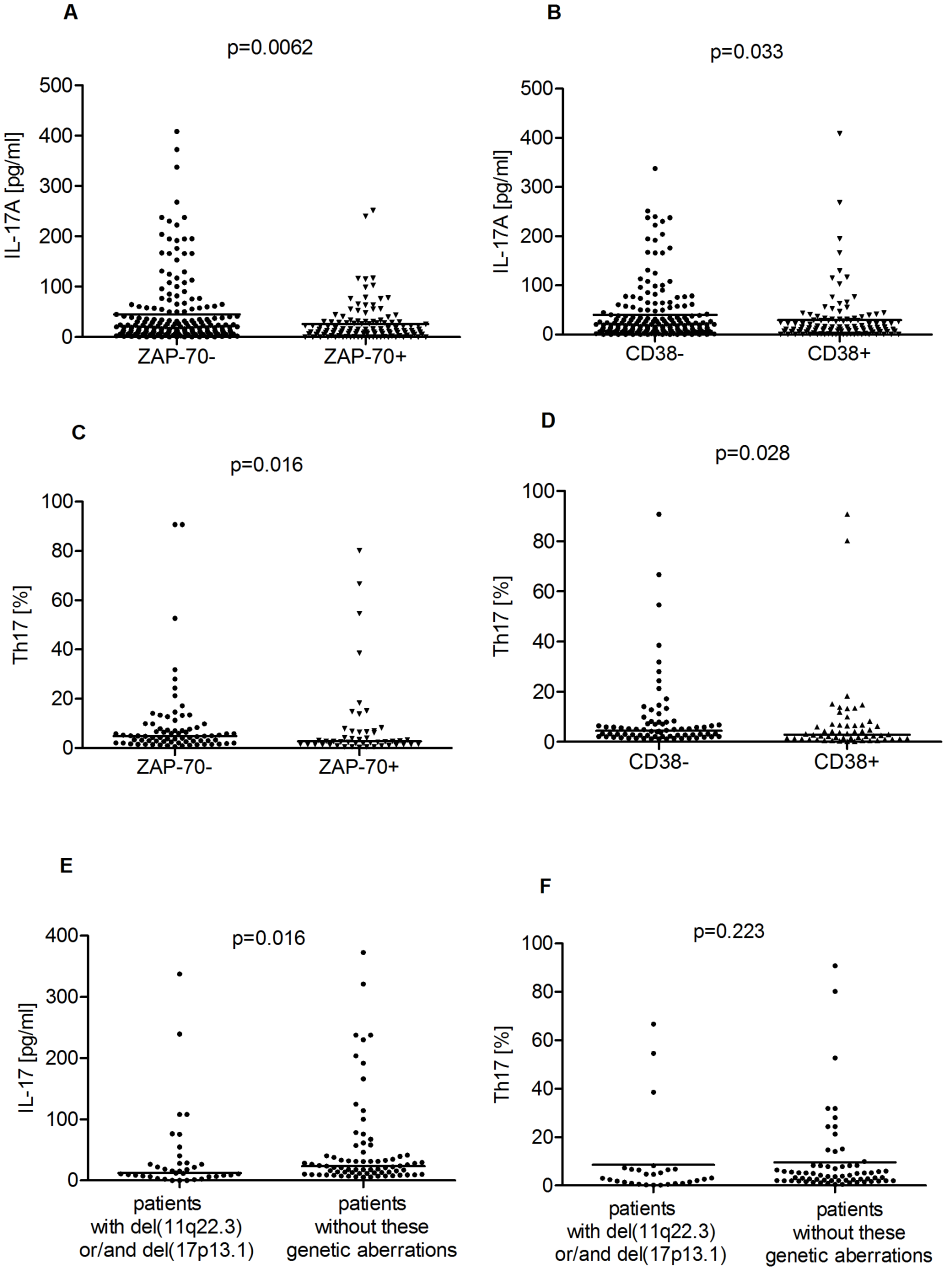
Plasma levels of IL-17A and percentage of Th17 cells in CLL patients analyzed by adverse prognostic factors. (A) Median plasma level of IL-17A in ZAP-70-negative patients in comparison to ZAP-70-positive patients (45.36 pg/ml vs. 26.11 pg/ml, p = 0.0062) (B). Median plasma level of IL-17A in CD38-negative patients and CD38-positive patients (39.73 pg/ml vs. 29.25 pg/ml, p = 0.033). (C) The percentage of CD4^+^/CD3^+^/IL-17A^+^ cells in ZAP-70-negative patients compared with ZAP-70-positive patients (4.85% vs. 2.67%, p = 0.016). (D). Median percentage of CD4^+^/CD3^+^ cells with intracellular IL-17A expression in CD38-negative and CD38-positive patients (4.46% vs. 2.87%, p = 0.028). (E,F) Plasma levels of IL-17 and percentage of Th17 cells in CLL patients, subdivided according to cytogenetic analysis. (E) median levels of IL-17A in CLL patients carrying the 11q22.3 deletion and/or the 17p13.1 deletion (12.24 pg/ml) and patients without these genetic aberrations (23.55 pg/ml; p = 0.016) (F) Th17 cell percentages in patients with del(11q22.3) or/and del(17p13.1) and patients without these unfavorable genetic aberrations (8.55% vs. 9.65%).

The IL-17A concentration in PB was significantly higher in CLL patients than in HV (39.35 pg/ml vs. 14.94 pg/ml, p = 0.0018) (Figure 2A). There were no significant differences in IL-17A plasma levels between PB and BM (39.35 pg/ml vs. 37.10 pg/ml) in CLL patients (p = 0.059). The plasma level of IL-17A inversely correlated with the stage of disease (R = −0.259; p = 0.006). CLL patients with Rai Stage 0 had significantly higher levels of IL-17A (median: 44.63 pg/ml) than those with Rai Stages I–II (median: 35.33 pg/ml) or III–IV (median: 15.23 pg/ml) (Figure 3A). A significantly higher median plasma IL-17A level was observed in ZAP-70-negative patients in comparison to ZAP-70-positive patients (45.36 pg/ml vs. 26.11 pg/ml, p = 0.0062) (Figure 4A). Likewise, we observed a significantly higher plasma IL-17A level in CD38-negative patients in comparison to CD38-positive patients (39.73 pg/ml vs. 29.25 pg/ml, p = 0.033) (Figure 4B). The plasma levels of IL-17A inversely correlated with the levels of TNF (R = −0.286; p = 0.039) and IL-10 (R = −0.551; p = 0.0001).

### Intracellular IL-17A Expression

In CLL patients as well as in HV, the percentage of CD4^+^/CD3^+^/IL-17A^+^ (Th17) cells with intracellular IL-17A expression in non-activation assays was frequently lower than 1%, comparable with the level of autofluorescence. Despite the fact that IL-17A mRNA was not detected in T cells of the majority of CLL patients, the IL-17A protein was present in T cells after PMA and ionomycin stimulation. We found a significantly higher median percentage of CD4^+^/CD3^+^/IL-17A^+^ cells in CLL patients than in HV (8.76% vs. 3.02%, p = 0.035) (Figure 2B). In the CLL patient group, there was a significant correlation between Th17 cell percentage and IL-17A plasma levels (R = 0.202; p = 0.036). There were no significant differences in Th17 cell percentages between PB and BM (8.76% vs. 9.96%) in CLL patients (p = 0.056). The expression of IL-17A in CD4^+^/CD3^+^ cells showed low interindividual variability in HV. In CLL patients, IL-17A expression was more diverse and significantly higher in patients in Rai Stage 0 (median, 12.11%) as compared to those in Rai Stages I–II (median, 8.11%) and III–IV (median, 5.15%) (Figure 3B). Additionally, the percentage of CD4^+^/CD3^+^/IL-17A^+^ cells was significantly higher in ZAP-70-negative patients compared with ZAP-70-positive patients (4.85% vs. 2.67%) (Figure 4C). Likewise, a significantly higher percentage of CD4^+^/CD3^+^ cells with intracellular IL-17A expression in CD38-negative than in CD38-positive patients was observed (4.46% vs. 2.87%) (Figure 4D). Furthermore, there was a significant correlation between the percentages of Th17 and iNKT cells (R = 0.203; p = 0.036) and inverse correlations between the percentage of Th17 and Treg cells (R = −0.22; p = 0.049). Th17 cell percentages adversely correlated with IL-4^+^ producing Th2 cells (R = −0.35; p = 0.045) and CD4^+^ T cell producing TNF (R = −0.278; p = 0.039). The percentage of Th17 cells inversely correlated with β2-microglobulin serum levels (R = −0.29; p = 0.049).

### Expression of IL-17A mRNA in CD4^+^ T cells

Detectable amounts of IL-17A mRNA have been found in 5 out of CLL samples, whereas none of CD4+ T cells in the samples obtained from HV contained IL-17A mRNA. All CLL patients with detectable IL-17A mRNA in T cells were in Rai Stage 0 and negative both for ZAP-70 and CD38 expression. Each sample was normalized to GAPDH. In contrast, all CD4+ cells after PMA and ionomycin stimulation were found to express IL-17A mRNA (2^−ΔCT^ = 3.96; range, 0.5–14.6). The presence of IL-17A mRNA in CD4^+^ lymphocytes prompted us to analyze intracellular IL-17A expression.

### IL-17A Levels and Th17 Cell Percentage in Patients Carrying Unfavorable Cytogenetic Abnormalities

Karyotypic analysis at the time of testing was available for 124 out of the 294 study patients. There was a significant difference in median IL-17A levels between patients carrying the 11q22.3 deletion and/or the 17p13.1 deletion (12.24 pg/ml) and patients without these genetic aberrations (23.55 pg/ml; p = 0.016) (Figure 4E). There were no significant differences in Th17 cell percentages between patients with del(11q22.3) or/and del(17p13.1) and patients without these unfavorable genetic aberrations (8.55% vs. 9.65%) (Figure 4F).

### IL-17A-secreting Th17 Cells and Clinical Outcome of CLL Patients

During the follow-up period (median, 9 months; range, 0.5–123 months), 141 patients (47.95%) were treated, fulfilling the criteria of therapy initiation according to IWCLL [18]. Sixty-two patients were treated with chemo- or chemoimmunotherapy with fludarabine combined with cyclophosphamide (FC) or with cyclophosphamide and rituximab (FCR). Complete hematological remission (CR) was achieved in 32 patients (22.70%), partial remission (PR) in 41 patients (29.08%), and stable disease and disease progression were observed respectively in 34 (24.11%) and 34 (24.11%) of the patients.

The plasma level IL-17A measured at the time of diagnosis was significantly lower in patients requiring therapy (median, 11.22 pg/ml) as compared to patients without treatment during the observation period (median, 20.52 pg/ml) (p = 0.0003). Moreover, plasma levels of IL-17A correlated with the time from CLL diagnosis to the start of therapy (p = 0.027).

**Figure 5:**
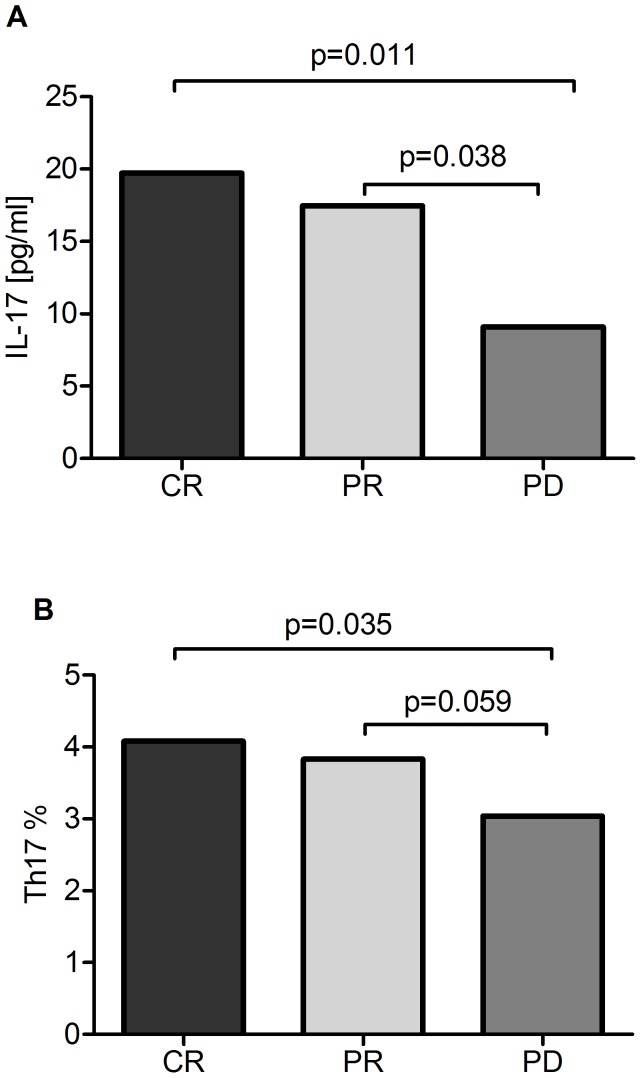
Plasma IL-17A levels and percentage of Th17 cells in treatment patients. (A) Plasma levels of IL-17A in patients who achieved hematological remission (CR: 19.73 pg/ml and PR: 17.47 pg/ml) after fludarabine-containing protocols in comparison to non-responding patients (PD: 9.09 pg/ml). (B) percentages of Th17 cells in the patients with progressive disease (3.04%) and those with complete (4.08%) or partial remission (3.83%). CR – complete remission; PR - partial remission; PD – progressive disease.

Patients who achieved hematological remission after fludarabine-containing protocols showed a higher plasma IL-17A level as compared to non-responding patients (Figure 5A). In the patients with progressive disease, the percentage of Th17 cells was significantly lower than in those with complete or partial remission (Figure 5B). Plasma levels of IL-17A (median, 16.94 pg/ml) were significantly lower in patients who died in contrast to surviving patients (median, 37.81 pg/ml) (p = 0.028).

We analyzed Kaplan-Meier curves for the overall survival (OS) in groups with high and low expression levels of IL-17A. Neither IL-17A level nor Th17 percentage has prognostic value for OS.

## Discussion

The role of the immune system in cancer development is undoubtedly crucial, though still not fully understood. Recently discovered novel subpopulations of Th cells, T regulatory cells, and Th17 cells have raised questions on their putative contribution on tumor immunity. In this study, we found both higher median percentage of PB CD4+ T-cells with intracellular expression of IL-17A and higher IL-17A plasma level in patients with CLL comparing to the healthy control that might suggest the potential role of IL-17 and Th17 cells in CLL immunobiology. This data is in agreement with the recent paper of Jain et al., who also reported higher absolute counts and percentages of Th17 cells in peripheral blood in CLL patients than in healthy controls [17]. High frequencies of Th17 cells and IL-17A levels in tumor tissues, malignant ascites fluid, and PB have been previously observed in patients with solid tumors [22]–[27] as well as hematological malignancies such as acute myeloid leukemia [28], myelodysplastic syndrome [29], and multiple myeloma [30], where it correlated with markedly more favorable clinical outcomes. However, it is possible that Th17 function may vary according to cancer cause, type and location as well as stage of disease, since collective evidence suggests that Th17 cells, may induce inflammation and promote the initiation and early growth of some tumors [31], [32]. In the current study, we observed significantly higher percentages of Th17 cells in patients in early clinical stages of CLL as compared to those in advanced Rai stages. It is possible, that Th17 cells might be one of the elements of the immune system responsible for protection from the proliferation of B cell leukemic clones, allowing to maintain control in early clinical stages.

In further analysis, we found inverse relationships between IL-17A-secreting Th cells and a progressive clinical course of CLL. Th17 percentages in PB were lower in patients who died due to CLL during the follow-up period as compared to surviving patients. IL-17A plasma levels were lower in patients who required therapy due to CLL during the follow-up period as compared to patients who were not treated, and correlated with the time from CLL diagnosis to the start of therapy. Th-17 frequencies and IL-17A plasma levels were significantly higher in patients with adverse prognostic factors such as 17p and/or 11q deletion and CD38 and ZAP-70 expression. All CLL patients with detectable IL-17 mRNA in T cells were negative for both ZAP-70 and CD38 expression. The percentage of Th17 cells was significantly lower in patients not responding to the first-line therapy with fludarabine-based regimens as compared to responding patients, suggesting its potential predictive value. The data are in agreement with the earlier observations of Jain et al. [17], who noted a correlation between Th17 cell count and overall survival in patients with CLL. Likewise Jadidi-Niaragh et al. [33] showed a lower frequency of Th17 cells in progressive CLL patients compared to indolent patients and normal subjects. The mechanisms, i.e. how the anti-tumor response is attenuated when the leukemic clone gains the advantage, remain unknown. Observations on the higher prevalence of Th17 cells in tumor tissue in early versus advanced clinical stages concerned solid tumors like ovarian [23], breast [24], and gastric [25] cancers where the Th17 cell percentages gradually decreased along with the progression of cancer.

The premise on the beneficial role of Th17 cells in anti-tumor immunity in CLL might be further supported by the positive correlation between Th17 and iNKT cells and adverse correlation between Th17 and Treg cells. It is important to note that iNKT cells have recently been revealed as a one of key players in the immune responses against tumors [34] and their numbers and function were found to be normal in patients with CLL in early stages [35]. Our results suggest that that progression of CLL is associated with downregulation of Th17 cells and expansion of Treg cells. It seems that Th17 cells are positively associated with effector cells and negatively associated with Treg. These data are in agreement with the recent paper of Jadidi-Niaragh et al. [33] who reported that the decrease in IL-17-producing T cells was associated with CD39^+^ Treg cells expansion. Adverse relationships between Th17 and T regulatory cells were described in the microenviroment of solid tumors such as breast and gastric cancer. In early clinical stages, the percentage of Th17 cells was significantly higher as compared to advanced disease, in contrast to Treg that accumulated along with disease progression [24], [25]. Moreover, T regulatory cells inhibit generation and differentiation of Th17 cells in malignant pleural effusion of patients with lung cancer [36]. Both populations seem to exert reverse effects on tumor immunity, analogously to autoimmune inflammation, where Th17 and Treg cells play critical roles and their mechanisms of action are opposite to one another [37]. Moreover, Th17 cell percentages adversely correlated with IL-4+ producing Th2 cells and CD4+T cell producing TNF, and the plasma level of IL-17A inversely correlated with the levels of TNF and IL-10. A decrease in the percentage of Th17 cells was accompanied by an increasing percentage of Th2 cells, which, similarly to T regulatory cells, is responsible for the induction of immune tolerance to cancer. All three cytokines are considered to play an important role in the CLL progression [38]. IL-4 protects leukemic cells from apoptosis by increasing expression of Bcl-2 [39] and creating a microenvironment favourable for their development. TNF is believed to inhibit apoptosis in leukemic B cells via the activation of NF-κB [40]. TNF levels in peripheral blood of CLL patients was found to be higher than in healthy subjects, increasing along with the stage of disease and correlating with a more aggressive clinical course [41], [42]. IL-10 not only directly suppresses immune responses, but is also produced by malignant B cells, being their autocrine growth factor via the ability to inhibit the induction of apoptosis [43]. IL-10 serum levels are significantly increased in CLL patients and are associated with poor prognosis [44].

The direct mechanism of Th17 action in CLL has not been yet studied and remain unexplained. The results of this study, suggest the beneficial role of Th17 in CLL immunity and indicate the need of further studies, not only in the context of their significance in the biology of this malignancy, but also the novel target for cellular immunotherapy.

## References

[1] Furman RR (2010) Prognostic markers and stratification of chronic lymphocytic leukemia. Hematology Am Soc Hematol Educ Program 2010: 77–81. 21239774 10.1182/asheducation-2010.1.77

[2] Tinhofer I, Rubenzer G, Holler C, Hofstaetter E, Stoecher M, et al (2006) Expression levels of CD38 in T cells predict course of disease in male patients with B-chronic lymphocytic leukemia. Blood 108: 2950–2956. 16825496 10.1182/blood-2006-03-010553

[3] Mellstedt H, Choudhury A (2006) T and B cells in B-chronic lymphocytic leukaemia: Faust, Mephistopheles and the pact with the Devil. Cancer Immunol Immunother 55: 210–220. 15906026 10.1007/s00262-005-0675-4 PMC11029856

[4] Palmer S, Hanson CA, Zent CS, Porrata LF, Laplant B, et al (2008) Prognostic importance of T and NK-cells in a consecutive series of newly diagnosed patients with chronic lymphocytic leukaemia. Br J Haematol. 141: 607–614. 10.1111/j.1365-2141.2008.07070.x PMC3840945 18384436

[5] Scrivener S, Goddard RV, Kaminski ER, Prentice AG (2003) Abnormal T-cell function in B-cell chronic lymphocytic leukaemia. Leuk Lymphoma 44: 383–389. 12688308 10.1080/1042819021000029993

[6] Podhorecka M, Dmoszynska A, Rolinski J, Wasik E (2002) T type 1/type 2 subsets balance in B-cell chronic lymphocytic leukemia–the three-color flow cytometry analysis. Leuk Res 26: 657–660. 12008083 10.1016/s0145-2126(01)00194-1

[7] Sasada T, Kimura M, Yoshida Y, Kanai M, Takabayashi A (2003) CD4+CD25+ regulatory T cells in patients with gastrointestinal malignancies: possible involvement of regulatory T cells in disease progression. Cancer 98: 1089–1099. 12942579 10.1002/cncr.11618

[8] Curiel TJ, Coukos G, Zou L, Alvarez X, Cheng P, et al (2004) Specific recruitment of regulatory T cells in ovarian carcinoma fosters immune privilege and predicts reduced survival. Nat Med 10: 942–949. 15322536 10.1038/nm1093

[9] Beyer M, Schultze JL (2009) Regulatory T cells: major players in the tumor microenvironment. Curr Pharm Des 15: 1879–1892. 19519430 10.2174/138161209788453211

[10] Beyer M, Kochanek M, Darabi K, Popov A, Jensen M, et al (2005) Reduced frequencies and suppressive function of CD4+CD25hi regulatory T cells in patients with chronic lymphocytic leukemia after therapy with fludarabine. Blood 106: 2018–2025. 15914560 10.1182/blood-2005-02-0642

[11] D’Arena G, Laurenti L, Minervini MM, Deaglio S, Bonello L, et al (2011) Regulatory T-cell number is increased in chronic lymphocytic leukemia patients and correlates with progressive disease. Leuk Res 35: 363–368. 20880586 10.1016/j.leukres.2010.08.010

[12] Gaffen SL (2008) An overview of IL-17 function and signaling. Cytokine 43: 402–407. 18701318 10.1016/j.cyto.2008.07.017 PMC2582446

[13] Khader SA, Gaffen SL, Kolls JK (2009) Th17 cells at the crossroads of innate and adaptive immunity against infectious diseases at the mucosa. Mucosal Immunol 2: 403–411. 19587639 10.1038/mi.2009.100 PMC2811522

[14] Gaffen SL (2009) The role of interleukin-17 in the pathogenesis of rheumatoid arthritis. Curr Rheumatol Rep 11: 365–370. 19772832 10.1007/s11926-009-0052-y PMC2811488

[15] Ji Y, Zhang W (2010) Th17 cells: positive or negative role in tumor? Cancer Immunol Immunother 59: 979–987. 20352428 10.1007/s00262-010-0849-6 PMC11031007

[16] Murugaiyan G, Saha B (2009) Protumor vs antitumor functions of IL-17. J Immunol 183: 4169–4175. 19767566 10.4049/jimmunol.0901017

[17] Jain P, Javdan M, Feger FK, Chiu PY, Sison C, et al (2012) Th17 and non-Th17 interleukin-17-expressing cells in chronic lymphocytic leukemia: delineation, distribution, and clinical relevance. Haematologica 97: 599–607. 22058222 10.3324/haematol.2011.047316 PMC3347674

[18] Hallek M, Cheson BD, Catovsky D, Caligaris-Cappio F, Dighiero G, et al (2008) International Workshop on Chronic Lymphocytic Leukemia. Guidelines for the diagnosis and treatment of chronic lymphocytic leukemia: a report from the International Workshop on Chronic Lymphocytic Leukemia updating the National Cancer Institute-Working Group 1996 guidelines. Blood 111: 5446–5456. 18216293 10.1182/blood-2007-06-093906 PMC2972576

[19] Rai KR, Sawitsky A, Cronkite EP, Chanana AD, Levy RN, et al (1975) Clinical staging of chronic lymphocytic leukemia. Blood 46: 219–234.1139039

[20] Bojarska-Junak A, Rolinski J, Wasik-Szczepanek E, Kaluzny Z, Dmoszynska A (2002) Intracellular tumor necrosis factor production by T- and B-cells in B-cell chronic lymphocytic leukemia. Haematologica 87: 490–499. 12010662

[21] Hus I, Podhorecka M, Bojarska-Junak A, Rolinski J, Schmitt M, et al (2006) The clinical significance of ZAP-70 and CD38 expression in B cell chronic lymphocytic leukaemia. Ann Oncol 17: 683–690. 16524977 10.1093/annonc/mdj120

[22] Zhou P, Sha H, Zhu J (2010) The role of T-helper 17 (Th17) cells in patients with medulloblastoma. J Int Med Res 38: 611–619. 20515574 10.1177/147323001003800223

[23] Kryczek I, Banerjee M, Cheng P, Vatan L, Szeliga W, et al (2009) Phenotype, distribution, generation, and functional and clinical relevance of Th17 cells in the human tumor environments. Blood 114: 1141–1149. 19470694 10.1182/blood-2009-03-208249 PMC2723011

[24] Wang J, Cai D, Ma B, Wu G, Wu J (2011) Skewing the balance of regulatory T-cells and T-helper 17 cells in breast cancer patients. J Int Med Res 39: 691–701. 21819700 10.1177/147323001103900301

[25] Maruyama T, Kono K, Mizukami Y, Kawaguchi Y, Mimura K, et al (2010) Distribution of Th17 cells and FoxP3(+) regulatory T cells in tumor-infiltrating lymphocytes, tumor-draining lymph nodes and peripheral blood lymphocytes in patients with gastric cancer. Cancer Sci 101: 1947–1954. 20550524 10.1111/j.1349-7006.2010.01624.x PMC11159855

[26] Zhang JP, Yan J, Xu J, Pang XH, Chen MS, et al (2009) Increased intratumoral IL-17-producing cells correlate with poor survival in hepatocellular carcinoma patients. J Hepatol 50: 980–989. 19329213 10.1016/j.jhep.2008.12.033

[27] Liu J, Duan Y, Cheng X, Chen X, Xie W, et al (2011) IL-17 is associated with poor prognosis and promotes angiogenesis via stimulating VEGF production of cancer cells in colorectal carcinoma. Biochem Biophys Res Commun 407: 348–354. 21396350 10.1016/j.bbrc.2011.03.021

[28] Wu C, Wang S, Wang F, Chen Q, Peng S, et al (2009) Increased frequencies of T helper type 17 cells in the peripheral blood of patients with acute myeloid leukaemia. Clin Exp Immunol 158: 199–204. 19737137 10.1111/j.1365-2249.2009.04011.x PMC2768809

[29] Kordasti SY, Afzali B, Lim Z, Ingram W, Hayden J, et al (2009) IL-17-producing CD4(+) T cells, pro-inflammatory cytokines and apoptosis are increased in low risk myelodysplastic syndrome. Br J Haematol 145: 64–72. 19210506 10.1111/j.1365-2141.2009.07593.x

[30] Prabhala RH, Pelluru D, Fulciniti M, Prabhala HK, Nanjappa P, et al (2010) Elevated IL-17 produced by TH17 cells promotes myeloma cell growth and inhibits immune function in multiple myeloma. Blood 115: 5385–5392. 20395418 10.1182/blood-2009-10-246660 PMC2902136

[31] Wilke CM, Kryczek I, Wei S, Zhao E, Wu K, et al (2011) Th17 cells in cancer: help or hindrance? Carcinogenesis 32: 643–649. 21304053 10.1093/carcin/bgr019 PMC3086699

[32] Zou W, Restifo NP (2010) T(H)17 cells in tumour immunity and immunotherapy. Nat Rev Immunol 10: 248–256. 20336152 10.1038/nri2742 PMC3242804

[33] Jadidi-Niaragh F, Ghalamfarsa G, Memarian A, Asgarian-Omran H, Razavi SM, et al (2013) Downregulation of IL-17-producing T cells is associated with regulatory T cell expansion and disease progression in chronic lymphocytic leukemia. Tumour Biol 34: 929–940. 23269607 10.1007/s13277-012-0628-4

[34] Seino K, Motohashi S, Fujisawa T, Nakayama T, Taniguchi M (2006) Natural killer T cell-mediated antitumor immune responses and their clinical applications. Cancer Sci 97: 807–812. 16805854 10.1111/j.1349-7006.2006.00257.x PMC11158813

[35] Weinkove R, Brooks CR, Carter JM, Hermans IF, Ronchese F (2013) Functional invariant natural killer T-cell and CD1d axis in chronic lymphocytic leukemia: implications for immunotherapy. Haematologica 98: 376–384. 23065503 10.3324/haematol.2012.072835 PMC3659950

[36] Ye ZJ, Zhou Q, Zhang JC, Li X, Wu C, et al (2011) CD39+ regulatory T cells suppress generation and differentiation of Th17 cells in human malignant pleural effusion via a LAP-dependent mechanism. Respir Res 12: 77. 21663645 10.1186/1465-9921-12-77 PMC3120670

[37] de Jong E, Suddason T, Lord GM (2010) Translational mini-review series on Th17 cells: development of mouse and human T helper 17 cells. Clin Exp Immunol 159: 148–158. 19912248 10.1111/j.1365-2249.2009.04041.x PMC2810383

[38] Lech-Maranda E, Grzybowska-Izydorczyk O, Wyka K, Mlynarski W, Borowiec M, et al (2012) Serum tumor necrosis factor-α and interleukin-10 levels as markers to predict outcome of patients with chronic lymphocytic leukemia in different risk groups defined by the IGHV mutation status. Arch Immunol Ther Exp 60: 477–486. 10.1007/s00005-012-0197-7 22945689

[39] Dancescu M, Rubio-Trujillo M, Biron G, Bron D, Delespesse G, et al (1992) Interleukin 4 protects chronic lymphocytic leukemic B cells from death by apoptosis and upregulates Bcl-2 expression. J Exp Med 176: 1319–1326. 1402678 10.1084/jem.176.5.1319 PMC2119420

[40] Podhorecka M (2004) Apoptosis in pathogenesis of B-cell chronic lymphocytic leukemia. Postepy Hig Med Dosw 58: 236–242. 15224009

[41] Ferrajoli A, Keating MJ, Manshouri T, Giles FJ, Dey A, et al (2002) The clinical significance of tumor necrosis factor-alpha plasma level in patients having chronic lymphocytic leukemia. Blood 100: 1215–1219. 12149200

[42] Bojarska-Junak A, Hus I, Szczepanek EW, Dmoszyńska A, Roliński J (2008) Peripheral blood and bone marrow TNF and TNF receptors in early and advanced stages of B-CLL in correlation with ZAP-70 protein and CD38 antigen. Leuk Res 32: 225–233. 17675228 10.1016/j.leukres.2007.06.007

[43] Yen Chong S, Lin YC, Czarneski J, Zhang M, Coffman F, et al (2001) Cell cycle effects of IL-10 on malignant B-1 cells. Genes Immun 2: 239–247. 11528515 10.1038/sj.gene.6363773

[44] Fayad L, Keating MJ, Reuben JM, O’Brien S, Lee BN, et al (2001) Interleukin-6 and interleukin-10 levels in chronic lymphocytic leukemia: correlation with phenotypic characteristics and outcome. Blood 97: 256–263. 11133769 10.1182/blood.v97.1.256

